# Study on the Re-Aging Behavior of Cu-Rich Precipitates in a FeCu Alloy under Electropulsing

**DOI:** 10.3390/ma17061287

**Published:** 2024-03-11

**Authors:** Shengjun Xia, Tinghe Yang, Menglin Gao, Xing Hu, Qiulin Li

**Affiliations:** 1 Institute of Materials Research, Shenzhen International Graduate School, Tsinghua University, Shenzhen 518055, China; 2 School of Materials Science and Engineering, Tsinghua University, Beijing 100084, China

**Keywords:** Cu-rich precipitates, re-aging, electropulsing, annealing, life extension

## Abstract

The nanoscale Cu-rich precipitates (CRPs) are one of the most critical microstructural features responsible for degrading the mechanical properties of reactor pressure vessel (RPV) steels. The prospect of the rapid regeneration of the service performance of degraded materials through electropulsing is attractive, and electropulsing has been proven to have the application potential to eliminate the CRPs and recover the mechanical properties of RPV materials. However, few studies have investigated the secondary service issue of electropulsing. This paper provides experimental findings from microstructural investigations and property evaluations of a FeCu RPV model alloy subjected to re-aging following recovery electropulsing and annealing treatments. The evolution behavior of CRPs and the changes in the hardness of the alloy during the re-aging process after electropulsing treatment were consistent with the initial aging process, while the re-aging process of the annealing treatment group was quite different from the initial aging. The difference between the electropulsing and annealing treatment groups was that the annealing treatment failed to eliminate the precipitates completely, leaving behind some large precipitates. This work demonstrates the potential application of EPT in this field.

## 1. Introduction

A reactor pressure vessel (RPV) is a unique and irreplaceable equipment that holds the coolant and reactor core in a nuclear power plant (NPP). However, the radiation-induced embrittlement and hardening of RPV steels are a factor of concern, which hinders the life extension of the RPV. Typically, nanoscale precipitates (mainly Cu-rich precipitates (CRPs) and MnNi-rich precipitates (MNPs)) and matrix damage (mainly dislocation loops and voids) are responsible for the degradation of the mechanical properties of RPV steels [1,2,3,4]. Specifically, CRPs contribute the most to the hardening of the RPV steels that were not intentionally controlled for Cu content used in early NPPs [3,5]. These early NPPs are currently facing the issue of the expiration of their lifespan. Given the role of the RPV in NPPs, its lifetime extension has become a feasible and economical choice for reactors approaching the design lifetime of 40 years.

Annealing treatment (AT) is applied to alleviate irradiation-induced precipitates and defects and recover RPV’s mechanical properties in the past few decades to extend the in-service lifetime of the RPV [6,7,8,9]. Unfortunately, this conventional method generally requires a high treatment temperature and long operation time, inevitably wasting considerable energy due to the huge size of the RPV. For example, in the study of Kuleshova et al. [10], the RPV was heated up to 475 °C for 150 h in the post-irradiation annealing. Furthermore, the issue of the secondary service after AT is still under study. Shi et al. [11] investigated the microstructures and hardness evolution in initial irradiated, post-irradiation annealed, and re-irradiated Chinese-type low-Cu RPV steel. They found that the hardness of the re-irradiated state was higher than that of the initial-irradiated state and inferred that it was due to defects that had not been eliminated by annealing. Kuleshova et al. [12] investigated the microstructural evolution of VVER-1000 RPV steel under the primary irradiation and re-irradiation. They found that the microstructures of the RPV steel after the primary irradiation and re-irradiation after recovery annealing up to the close values of neutron fluences were identical. Moreover, traditional heat treatment needs people to remove the core equipment from RPV and prepare special heating facilities. Therefore, finding a feasible in-situ technique that could eliminate these nanoscale defects and restore the mechanical properties of RPV with low energy consumption is desirable.

As an instantaneous high-energy input processing technology, the electropulsing (or pulsed electric current) technique has been widely used to regulate the microstructures and properties of metals. Numerous researchers have carried out a lot of electropulsing treatment (EPT) experiments in many scientific fields of materials, including recovery and recrystallization [13,14,15,16], phase transformation [17,18,19,20], and precipitates regulation [21,22,23,24]. Zhao et al. [25] and Liu et al. [26] used electropulsing to regenerate the microstructures of thermally aged duplex stainless steels and successfully reversed the performance degradation. The prospect of the rapid regeneration of the microstructures and service performance of degraded materials through electropulsing is very attractive. In previous studies, Gao et al. [27] and Qin et al. [28] independently treated FeCu model alloys or Cu-added commercial SA508-III steels with the EPT technique to dissolve the CRPs. Both studies confirmed that electropulsing could rapidly and efficiently dissolve the CRPs at lower temperatures than post-irradiation annealing. However, the relevant studies mentioned earlier did not consider the secondary service of materials after performance regeneration through EPT. Will the performance degradation of materials during the secondary service become faster after undergoing performance regeneration? It is necessary for people to understand the secondary service performance of materials after EPT before the technique is put into practical use.

EPT has been demonstrated to efficiently restore the performance of RPV model materials by regulating CRPs. Whether its advantages over traditional annealing can be extended to the field of secondary service remains to be studied. It is natural to investigate the evolution behavior of CRPs in RPV steels or its model materials during secondary service. Obtaining CRPs through irradiation is complex and challenging, so thermally aged FeCu alloys are usually used to simulate irradiated RPV steels [29,30,31]. Therefore, this work focuses on the re-aging behavior of CRPs in a FeCu model alloy under electropulsing, which could support the reliability of EPT being applied to RPV degradation recovery.

## 2. Materials and Methods

The FeCu model alloy used in this study was ordered from the China Central Iron & Steel Research Institute, Beijing, China. The nominal chemical composition of the model alloy is Fe-1.1 wt.% Cu, as shown in Table 1. The as-received (AR) alloy was solution-treated at 880 °C for 12 h and then water-quenched. Subsequently, the AR alloy was cut into rectangular samples (50 × 10 × 2 mm^3^) for the following experiments. The samples in this state were labeled as ST ones.

**Figure 1:**
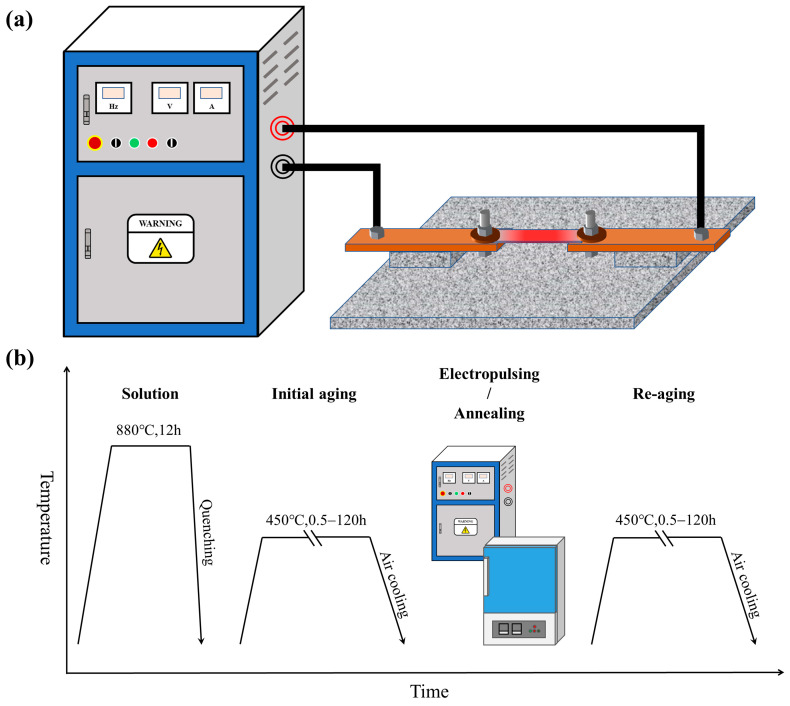
The schematic diagrams of (**a**) the EPT platform and (**b**) the treatment processes.

The ST samples were isothermally heated at 450 °C for different durations, and the aging treatment parameters selected for this study are shown in Table 2. The parameters used in the initial aging and re-aging processes were identical. The EPT and AT were applied to the samples initially aged for 100 h (IA-100h state, the reasons for parameter selection will be discussed in Section 3.1) to restore the microstructure and properties of the FeCu alloy. The EPT was conducted through a processing platform developed by the research team, as shown in Figure 1a. A AZ9881 thermometer (AZ Instrument, Taichung, China) with a K-type thermocouple clamped on the surface of the samples by a ceramic tweezer was used to record the temperature accurately during the treatment. The electric parameters were measured with a BH-0.66 current transformer (CHINT, Wenzhou, China) and a Tektronix TBS 1102B-edu electronic oscilloscope (Tektronix, Shanghai, China). The parameters during the EPT process are presented in Table 2, and the temperature column represents the highest temperature recorded by the thermometer. 

For comparison, some samples in the IA-100h state were heated in a muffle furnace, and the annealing temperature was the same as the EPT samples. The AT parameters are also shown in Table 2 The samples annealed for 5 h (AT-5h state) were chosen to undergo the following re-aging treatment, and the reasons for this will be introduced in Section 3.1. After EPT or AT, the samples underwent re-aging treatment, with the processing parameters identical to those of the initial aging treatment, as shown in Table 2. The experiment processes of the FeCu alloy in this work are shown in Figure 1b.

The Vickers hardness tests were conducted on a HVS-1000B Vickers microhardness tester (Laizhou Huayin Testing Instrument, Laizhou, China) under a load of 300 gf for 10 s, according to GB/T 4340.1-2009. A 10 × 10 × 2 mm^3^ section was cut from the central part of each sample for microhardness tests. The hardness specimens were successively polished with 400, 600, 1000, 1500, and 2000# sandpaper before being polished with 0.5 μm diamond polishing liquid. The measurements were taken at ten random positions for each specimen. The electrical resistivity was measured at 25 °C using a PPMS-9 (Quantum Design, San Diego, CA, USA) physical property measurement system in accordance with the requirements of GB/T 351-2019. Strip specimens of 2 × 2 × 10 mm^3^ were cut from the samples and then polished with 2000# sandpaper for electrical resistivity tests. The morphology and distribution of the CRPs were characterized using a JEM-3200FS field emission transmission electron microscopy (TEM) operated at 300 kV equipped with the scanning transmission electron microscopy (STEM) mode (JEOL, Tokyo, Japan). The TEM specimens were initially mechanically thinned to a thickness of 40 to 50 μm using SiC sandpaper, followed by further thinning through electro-polishing using an electrolyte of 10% perchloric acid and 90% ethanol in a RL-2 double-jet electro-polisher (Smart Innovate, Beijing, China) at −20 °C.

## 3. Results

### 3.1. Hardening Behavior

**Figure 2:**
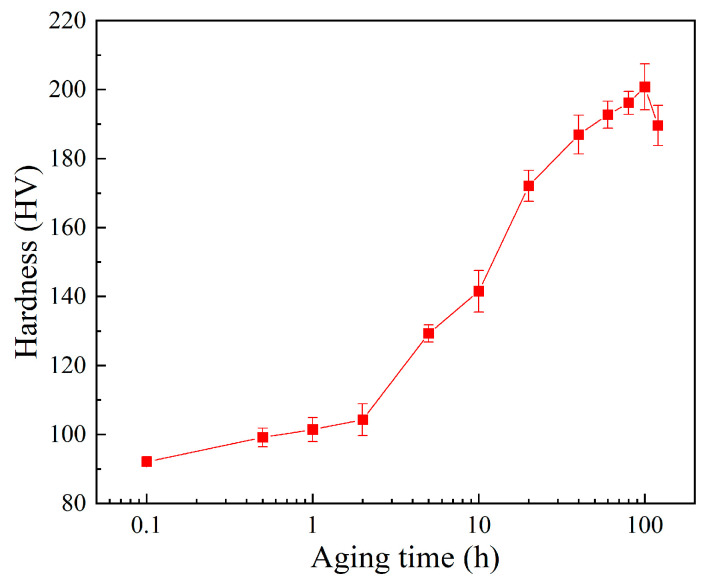
Variation of Vickers hardness with aging time during the initial aging process.

The variation of Vickers hardness with aging time during the initial aging process shown in Figure 2 demonstrates a general hardening–softening trend during the aging process attributed to precipitation hardening mechanisms [32]. The hardness peaked at 201 HV after aging for 100 h and subsequently began to decrease, and subsequent EPT and AT processes were performed based on the samples in the IA-100h state. This phenomenon is similar to the research findings of Jung et al. [33] on a copper-containing carbon steel and Li et al. [34] on a FeCu alloy, and it is attributed to the precipitation strengthening effect of CRPs [35].

**Figure 3:**
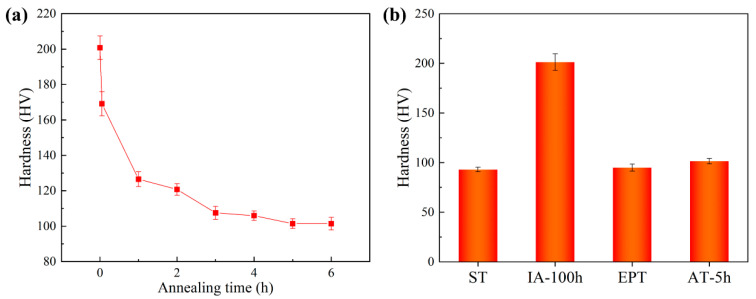
(**a**) Variation of Vickers hardness with annealing time. (**b**) Vickers hardness of different states.

Figure 3a shows the variation of Vickers hardness with annealing time. It is apparent that the hardness of the samples rapidly decreased with the prolonged annealing time and stabilized at approximately 101 HV after annealing for 5 h. Considering practical applications where shorter heat treatment times are typically preferred, a 5 h duration was selected as the processing parameter for the AT group before the re-aging treatment. The Vickers hardness of different states is shown in Figure 3b. It is worth noting that the hardness of samples treated with electropulsing (EPT, 95 HV) and 5 h duration annealing (AT-5h, 101 HV) has recovered to levels close to that of the solution state (ST, 93 HV) from the aged state (IA-100h, 201 HV). Considering the lower hardness of the EPT state and the shorter processing time of EPT, electropulsing exhibits better recovery effectiveness and higher processing efficiency than traditional heat annealing.

**Figure 4:**
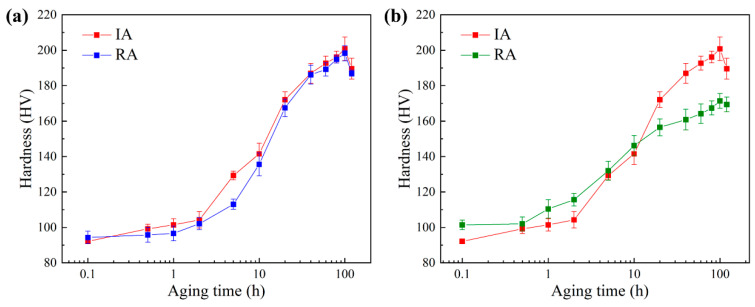
Variation of Vickers hardness with aging time during initial aging (IA) and re-aging (RA): (**a**) EPT group, (**b**) AT group.

The comparison of hardness variations during the re-aging and initial aging processes for the FeCu alloy treated with different recovery methods is illustrated in Figure 4. As evident from Figure 4a, during the re-aging process, the hardness of the EPT samples rapidly increased in a short period. At 20 h of aging, the hardness reached 167 HV. Subsequently, the rate of increase slowed down after 20 h, reaching its peak value of 198 HV at 100 h, followed by overaging, resulting in a decline in hardness. The trend of hardness variation during re-aging was generally consistent with the trend observed during the initial aging process for the EPT samples. As shown in Figure 4b, the hardness of the AT samples annealed for 5 h gradually increased with the aging time in the subsequent re-aging process. It reached around 156 HV at 20 h, and peaked at around 170 HV at about 100 h. Through comparison, the rate of hardness increase during re-aging was first faster and then slower than initial aging, and the peak hardness during re-aging did not exceed the peak hardness of initial aging. Specifically, the hardness growth of the AT group was significantly higher than that of the EPT group in the first 10 h during the re-aging process. The hardness of the AT group and EPT group after 10 h of re-aging was 146 HV and 136 HV, respectively. The differences in the hardness evolution trends of the EPT and AT group during the re-aging process require further analysis, combining observations on the evolution behavior of the CRPs.

### 3.2. Cu Precipitation Behavior

**Figure 5:**
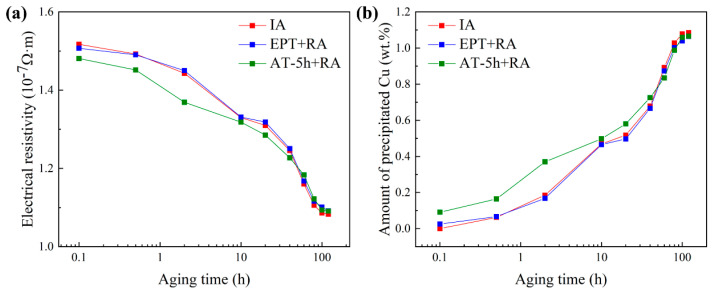
Variation of (**a**) electrical resistivity and (**b**) amount of precipitated Cu with aging time during initial aging (IA) and re-aging (RA).

Figure 5a shows the variation of electrical resistivity with aging time during initial aging and re-aging. With the precipitation of Cu atoms, the resistivity of the specimens gradually decreased with the prolongation of the aging time. The amount of the precipitated Cu atoms from the α-Fe matrix is often quantitatively obtained from the change in electrical resistivity based on the following equation [36]:

(1) $$\rho (n\Omega \cdot m)=\rho_{\mathrm{iron}}+340C+146N+135\mathrm{Si}+54\mathrm{Cr}+50\mathrm{Mn}+15\mathrm{Ni}+34\mathrm{Mo}+40\mathrm{Cu},$$

where each alloy element denotes the amount of a solute atom in weight percent, and ρ and ρ_iron_ are the electrical resistivity values of the specimen and pure iron at room temperature, respectively. According to Equation (1), a reduction of 40 nΩ·m in electrical resistivity is observed for every weight percent of Cu atoms precipitated from the matrix. The weight fraction of precipitated Cu atoms calculated from electrical resistivity is shown in Figure 5b. For the EPT samples, the precipitation amount and process of Cu atoms during initial aging and re-aging were very similar, with precipitation amounts reaching 1.08 wt.% and 1.04 wt.% at 100 h, respectively. However, for the AT samples, the precipitation process of Cu atoms during re-aging showed a first fast and then slow trend compared to initial aging. Specifically, for the AT-5h state (the origin of the AT-5h + RA curve in Figure 5a), its electrical resistivity was higher than that of the solution state (the origin of the IA curve in Figure 5a), corresponding to a Cu precipitation of 0.091 wt.%, accounting for approximately 10% of the total Cu atoms. This result is consistent with the hardness result, indicating that AT has not entirely eliminated the precipitated CRPs.

### 3.3. Observation of CRPs

**Figure 6:**
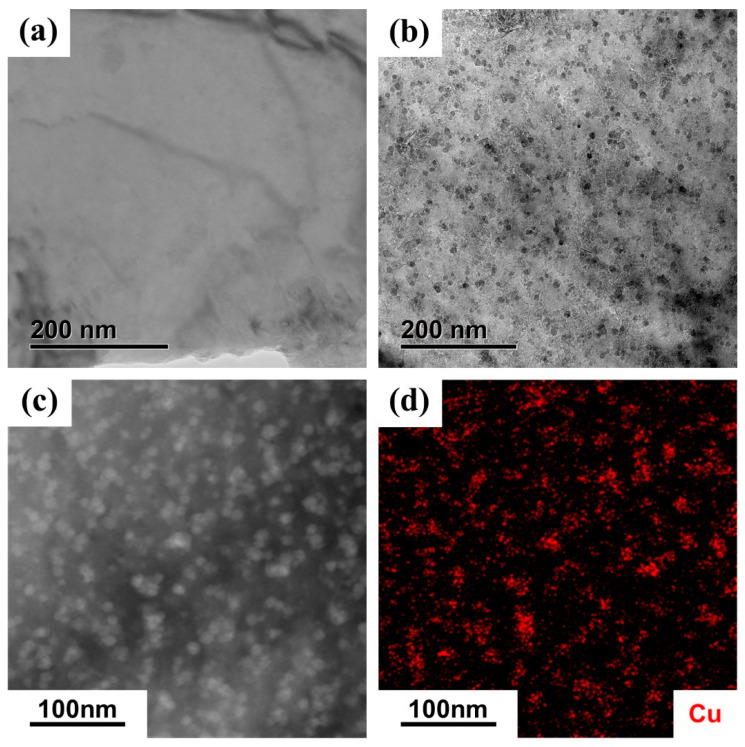
The TEM bright field image of (**a**) ST and (**b**) IA-100h states. The (**a**) high angle annular dark field (HAADF) image and (**d**) energy dispersive X-ray spectroscopy analysis of the precipitates in IA-100h state.

Figure 6 shows the TEM observation results of ST and IA-100h states. In Figure 6a,b, the ST specimen demonstrated no signs of precipitates, while many nanoscale dark precipitates with a particle size ranging from 5 to 15 nm formed in the α-Fe matrix after aging for 100 h. Overall, the CRPs appeared in a relatively uniform distribution, while some agglomerated precipitates were observed. The nanoscale precipitates were determined to be Cu-rich according to the results of high angle annular dark field (HAADF) image and elemental analysis, shown as Figure 6c,d.

**Figure 7:**
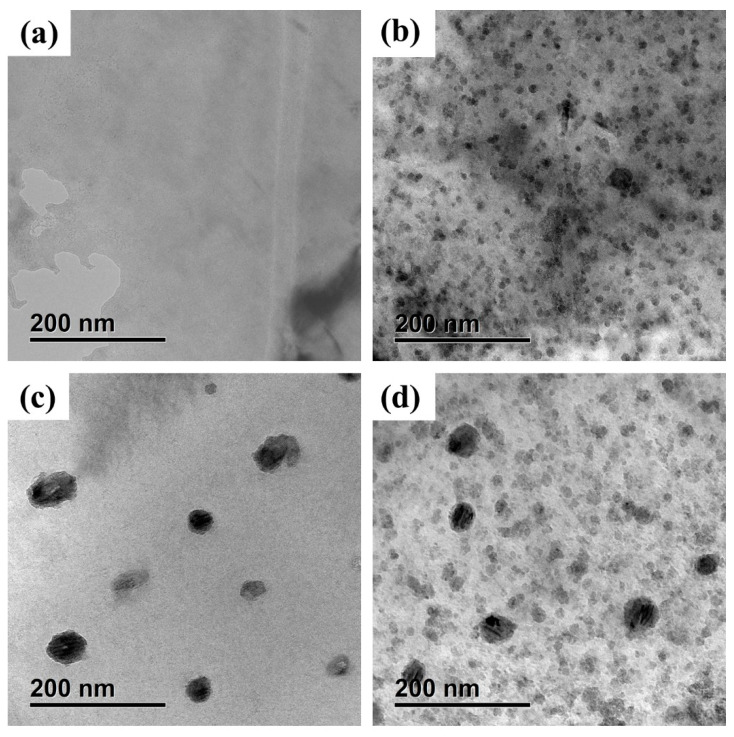
TEM bright field images of different states: (**a**) EPT; (**b**) EPT + RA-100h; (**c**) AT-5h; (**d**) AT-5h + RA-100h.

Figure 7 shows the TEM bright field images of the EPT and AT-5h samples before and after re-aging for 100 h (EPT/AT-5h + RA-100h states). Figure 7a shows the reverse evolution of the CRPs under EPT, as there were few obvious precipitates observed in the EPT specimen. After re-aging for 100h, a large number of CRPs were formed in the matrix, as shown in Figure 7b. The uniform distribution of CRPs in the EPT + RA-100h state was similar to that of the IA-100h state. Nevertheless, Figure 7c reveals that even after annealing for 5 h, a small number of large CRPs with a diameter of around 30 nm still existed in the AT-5h specimen. It can be concluded that EPT removed all the CRPs in the aged FeCu alloy, while AT only removed most of them. This is also consistent with the measure results from the hardness and electrical resistivity tests. According to Figure 7d, many CRPs appeared in the alloy matrix after re-aging for 100 h. However, the distribution of CRPs in the AT-5h + RA-100h state was very different from the IA-100h state, with two types of particles with different sizes. The large precipitates were residual CRPs observed in the AT-5h state, and the size of the small CRPs generated during the re-aging process was larger than that of the initial aging process.

**Figure 8:**
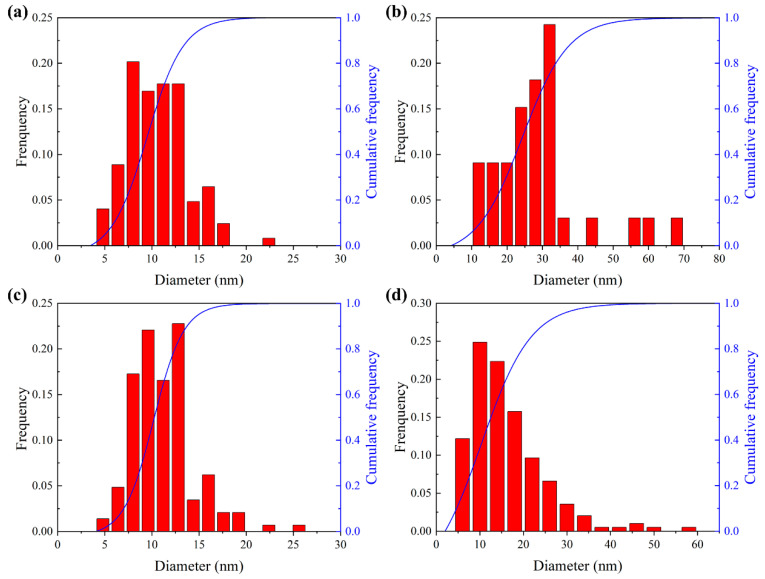
Size distributions of the CRPs in different states: (**a**) IA-100h; (**b**) AT-5h; (**c**) EPT + RA-100h; (**d**) AT-5h + RA-100h.

The size of the precipitates in this study was measured from the TEM bright field images using an image analysis software FIJI. The statistical results for the CRPs size distributions of different states are present in Figure 8. For the EPT process, the distribution of CRPs in the RA-100h state was similar to that of the IA-100h state, with the particle size concentrated in the range of 5 to 15 nm. The mean sizes of the CRPs in the IA-100h and EPT + RA-100h states were 10.5 ± 3.2 nm and 11.2 ± 3.5 nm. The number densities of the CRPs were approximated to be 1.24 × 10^22^ m^−3^ and 1.16 × 10^22^ m^−3^ under a reasonable assumption of an average foil thickness of 50 nm in the regions measured, respectively. The differences between the initial aging and re-aging can be ignored, considering the statistical errors. The mean precipitate sizes are in good agreement with the measured results obtained in FeCu model alloys under electron irradiation [37] and thermal aging [34]. For the AT process, the size distribution of CRPs in the RA-100h state exhibited a higher range compared to the IA-100h state. It is worth noting that the size of CRPs in the AT-5h + RA-100h state concentrated at 10 to 20 nm, while there were also a small number of large precipitates with a size of around 30 nm. The mean size and density of CRPs in the AT-5h + RA-100h state were 16.3 ± 8.6 nm and 0.78 × 10^22^ m^−3^. Compared to the initial aging, the average size of CRPs produced by re-aging increased by about 59%, while the density decreased by 37%. In particular, statistics on CRPs with a size larger than 20 nm in the AT-5h+RA-100h state showed that the mean size and density of the CRPs were 29.5 ± 9.0 nm and 0.12 × 10^22^ m^−3^, which were slightly higher than the statistical results of the AT-5h state (28.6 ± 12.9 nm, 0.10 × 10^22^ m^−3^). This indicates that the changes in residual CRPs during the re-aging process were insignificant. Combined with the results of electrical resistivity, the observation of CRPs indicates that the residual CRPs accelerated the nucleation, growth, and ripening of newly generated CRPs, increasing the mean size of CRPs. The smaller density of CRPs after ripening also decreased the peak hardness for the AT group (from 201 HV to 170 HV).

## 4. Discussion

The strengthening mechanism of CRPs can be explained by modulus strengthening based on the model developed by Russell and Brown [37]. The disparity in the shear modulus between CRPs and the α-Fe matrix leads to a difference in the dislocation energy between the two phases, ultimately enhancing the strength of the FeCu alloy by hindering the dislocation movement. According to the Russell and Brown model, the modulus strengthening effects are described as [37,38]:

(2) $$\sigma_{m}=1.15r^{\frac{1}{2}}N^{\frac{1}{2}}MGb{\left[1-{\left(\frac{E_{p}}{E_{m}}\right)}^{2}\right]}^{\frac{3}{4}},$$

where *M* = 3 is the Taylor factor, *G* = 80 Mpa is the shear modulus of the *α*-Fe matrix, *b* = 0.25 nm is the Burgers vector of dislocations in the *α*-Fe matrix, *N* is the density of precipitates, and *r* is the mean size of the precipitates. *E_p_* and *E_m_* are the dislocation line energy in the precipitate and matrix, respectively. *E_p_*/*E_m_* decreases as *r* increases. According to Equation (2), the precipitation hardening is closely related to the mean size and density of CRPs. In the early aging stage, *r* and *N* rapidly increased, causing the FeCu alloy to harden continuously. As Cu atoms precipitated close to saturation, the precipitates began to ripen, *N* rapidly decreased, and the hardness of the material reached its peak.

The solid solubility of Cu in bcc-Fe is less than 1 wt.% at 700 °C [39], so slow heat treatment following the thermodynamic phase diagram cannot entirely eliminate CRPs at this temperature. The CRPs in the AT samples were accompanied by Ostwald Ripening during the dissolution process, eventually forming large-sized residual CRPs [40,41]. Similar phenomena have also been reported in the hot annealing studies of Gurovich et al. [7] and Monzen et al. [42] In the present work, the different dissolution behavior of the CRPs in the α-Fe matrix has been observed after EPT and AT. The phase transformation can be described as *α* + CRPs → *α*′, where *α*′ is a supersaturated solid solution. According to classical thermodynamics, the requirement for precipitates’ dissolution is sufficient energy, which overcomes the energy barrier Δ*G*_0_ of inverse transformation. For inverse transformation in the thermodynamic equilibrium: *α* + CRPs → *α*′, Δ*G*_0_ can be written as [21,43]:

(3) $$\Delta G_{0}=A-B\cdot T,$$

where *A*, *B* > 0, and *T* is the absolute temperature. Then the phase transformation temperature *T*_0_ in a current-free situation can be determined by Δ*G*_0_ = 0:

(4) $$T_{0}=\frac{A}{B}$$

For the EPT condition, the free energy change Δ*G*_E_ of the dissolution of precipitates can be expressed as [21,44,45]:

(5) $$\Delta G_{E}=\Delta G_{0}+\Delta G_{\mathrm{ELEC}}$$

The transformation temperature $T_{S}^{E}$ under electropulsing can also be determined from $\Delta G_{E}=0$, as follows:

(6) $$T_{S}^{E}=\frac{A}{B}-\frac{\Delta G_{\mathrm{ELEC}}}{B}$$

According to reference, $\Delta G_{\mathrm{ELEC}}$ can be simplified as [46,47,48]:

(7) $$\Delta G_{\mathrm{ELEC}}=\mu_{0}g\Delta Vj^{2}\xi ,$$

where *g* is a positive geometric factor for coarse-grained materials, *μ*_0_ is the vacuum permeability, *j* is the current density, and Δ*V* is the volume of a precipitate nucleus. $\xi =(\sigma_{\alpha }-\sigma_{\mathrm{CRP}})/(\sigma_{\mathrm{CRP}}+2\sigma_{\alpha })$, $\sigma_{\alpha }$, and $\sigma_{\mathrm{CRP}}$ are the electrical conductivity of the α-Fe matrix and CRPs, respectively.

There are still no clear data about the electrical resistivity of CRPs. Previous studies indicated that there are distortion areas in the precipitates and their boundary, which consists of multiple elements [49,50]. Therefore, the resistance of the precipitates is slightly higher than that of the matrix due to a greater electron scattering, and results in ξ>0, hence $\Delta G_{\mathrm{ELEC}}>0$, according to Equation (6). Comparing Equation (6) with Equation (4), $T_{S}^{E}<T_{S}$ is derived, which means that when the EPT was applied to the aged alloy, the generation of electric Gibbs free energy reduced the thermodynamic barrier in the dissolution process of CRPs. Thus, the apparent dissolution temperature of the CRPs under the EPT is lower than AT. Therefore, the dissolution of CRPs in the EPT samples was more thorough than that in the AT samples.

The difference between the dissolution effect of EPT and AT caused a difference in the re-aging process between the two groups of experiments. EPT did not affect the secondary service performance of the FeCu alloy. The re-aging trend of the EPT group was consistent with the initial aging. However, the re-aging process of the AT group showed a faster trend compared to the initial aging process, especially in the early stage of aging. These residual CRPs will reduce the service life of the alloy after annealing repair and continue to accumulate in subsequent repair service cycles. Nevertheless, our work focused on the evolution of CRPs using a simplified model alloy based on actual RPV steels. EPT may impact other structural characteristics, such as carbides and bainites in RPV steels, affecting their secondary service performance. More comprehensive research is needed in this area. Specifically, our ongoing research has shown that EPT at excessively high temperatures led to rapid grain growth and decreased alloy hardness, which undoubtedly puts higher demands on the selection of electrical parameters. The advancement of EPT equipment level and organizational characterization technology will provide a new perspective for applying EPT technology.

## 5. Conclusions

In summary, the re-aging behavior of nanoscale Cu-rich precipitates in a FeCu model alloy under two different recovery treatments, electropulsing and annealing, was investigated. The cyclic aging-recovering-aging process showed that the different dissolution results of CRPs under different recovery treatments affected the subsequent re-aging behavior of CRPs and further affected the property changes of the alloy. The main findings of this study can be summarized as follows:

(1) The evolution behavior of CRPs and the changes in the hardness of the alloy during the re-aging process after EPT with 15.4 A/mm^2^ were consistent with the initial aging process. The peak hardness of initial aging and re-aging was 201 HV and 198 HV, respectively.
(2) The evolution behavior of CRPs and the changes in the hardness of the alloy during the re-aging process after annealing for 5 h showed a faster trend than the initial aging process, especially in the early stage. The peak hardness of initial aging and re-aging was 201 HV and 170 HV, respectively.
(3) The dissolution of CRPs in the EPT samples was more thorough than that in the AT samples. The residual CRPs in the AT samples accelerated the nucleation and ripening of new generated CRPs, causing an accelerated aging process and a decrease in peak hardness.
(4) From the perspective of hardness and CRP evolution, the degradation rate of the FeCu alloy after EPT during re-aging was not faster than that of initial aging.

Many reactors are approaching their design life (40 years) nowadays, and there is a strong demand for methods to delay and recover RPV degradation. This work demonstrates the potential application of EPT in this field. Further research is required to investigate and comprehend the evolutionary mechanism of other types of defects in RPV under electropulsing.

## Figures and Tables

**Table 1:** The nominal chemical composition of the FeCu model alloy in this study.

Element (wt. %)	Fe	Cu	C	ELSE
FeCu	Bal.	1.1	<0.005	<0.05

**Table 2:** The initial aging (IA), re-aging (RA), electropulsing (EPT), and annealing (AT) treatment parameters used in present study.

Parameters	Temperature (°C)	Processing Time	Electric Parameters
IA or RA	450	0.5 h/1 h/2 h/5 h/ 10 h/20 h/40 h/60 h/ 80 h/100 h/120 h	-
EPT	700	150 s	200 Hz/75 V/ 15.4 A/mm^2^
AT	700	150 s/1 h/2 h/3 h/ 4 h/5 h/6 h	-

## Data Availability

The data presented in this study are available upon reasonable request from the corresponding author. The data are not publicly available due to privacy.
